# Human kallikrein gene 13 (KLK13) expression by quantitative RT–PCR: an independent indicator of favourable prognosis in breast cancer

**DOI:** 10.1038/sj.bjc.6600283

**Published:** 2002-05-06

**Authors:** A Chang, G M Yousef, A Scorilas, L Grass, P Sismondi, R Ponzone, E P Diamandis

**Affiliations:** Department of Pathology and Laboratory Medicine, Mount Sinai Hospital, Toronto, Ontario, Canada; Department of Laboratory Medicine and Pathobiology, University of Toronto, Toronto, Ontario, Canada; National Center of Scientific Research ‘Demokritos’, IPC, Athens, 153 10 Greece; Academic Division of Gynecological Oncology, University of Turin, Mauriziano Umberto Hospital and Institute for Cancer Research and Treatment (IRCC) of Candiolo, Turin, Italy

**Keywords:** kallikreins, breast cancer, serine proteases, quantitative PCR, human kallikrein 13, prognostic and predictive markers

## Abstract

Kallikreins are a group of serine proteases with diverse physiological functions. KLK13 (previously known as KLK-L4) is a novel kallikrein gene located on chromosome 19q13.4 and shares a high degree of homology with other kallikrein family members. Many kallikrein genes were found to be differentially expressed in various malignancies, and their regulation is controlled by steroid hormones in prostate and breast cancer cell lines. We studied the expression of KLK13 by quantitative reverse transcriptase–polymerase chain reaction in 173 patients with epithelial breast carcinoma. An optimal cutoff point equal to the 40th percentile was defined, based on the ability of KLK13 to predict disease-free survival. KLK13 values were then associated with other established prognostic factors and with disease-free survival and overall survival. Higher positivity for KLK13 expression was found in older, oestrogen receptor positive patients. In univariate analysis, KLK13 expression is a significant predictor of improved disease-free survival and overall survival (*P*<0.001 and *P*=0.009, respectively). Cox multivariate analysis indicated that KLK13 was an independent prognostic variable in the subgroups of patients with Grade I–II tumours and in patients who were oestrogen receptor and progesterone receptor positive, and node positive. Hazard ratios derived from Cox analysis, related to disease-free survival and overall survival were 0.22 (*P*=0.001) and 0.24 (*P*=0.008), respectively, for the Grade I–II group; 0.36 (*P*=0.008) and 0.44 (*P*=0.038), respectively, for the node positive group and 0.36 (*P*=0.008) and 0.18 (*P*=0.008), respectively, for the oestrogen receptor positive group. The adjusted hazard ratio for progesterone receptor positive patients for disease-free survival was 0.25 (*P*=0.012). For patients in the node positive and oestrogen receptor positive subgroup (*n*=51) the adjusted hazard ratio was 0.25 (*P*=0.006) and for the node positive and progesterone receptor positive subgroup (*n*=46) the hazard ratio was 0.24 (*P*=0.008). Taken together, these data suggest that higher KLK13 expression in these subgroups of breast cancer patients is associated with an approximately 55 to 80% reduction in the risk of relapse or death. We conclude that KLK13 expression, as assessed by quantitative reverse transcriptase–polymerase chain reaction, is an independent favourable prognostic marker for breast carcinoma.

*British Journal of Cancer* (2002) **86**, 1457–1464. DOI: 10.1038/sj/bjc/6600283 www.bjcancer.com

© 2002 Cancer Research UK

## 

Breast cancer is the most common malignancy affecting women. It is estimated that in 2001, about 40 000 women will die from recurring or metastatic breast cancer (Greenlee *et al*, 2001). Response to treatment through the course of this disease varies greatly (Buzdar, 2001). Metastasis requires certain interactions among breast cells, stroma and surrounding normal tissues, and it involves a variety of growth factors and adhesion molecules (Teicher, 1995). As to whether breast cancer is a disease that can spread systemically from its earliest stages or whether tumours must mature in size before metastasis is still controversial (Harris and Hellman, 1996). There is a need for systemic hormonal therapy and chemotherapy, even in local disease, to prevent progression to metastasis (Buzdar, 2001).

Since breast carcinomas show great variability in their biological and clinical behaviour, the need for reliable prognostic parameters is critical. Classical prognostic factors in primary breast carcinoma include tumour size, nodal status, age, histopathology nuclear grading to steroid hormone receptors (Lopez-Otin and Diamandis, 1998). Ploidy and proliferative capacity (S phase fraction) are two other well-characterised prognostic factors (Anbazhagan *et al*, 1991). All these prognostic factors have been shown to predict disease-free survival (DFS) and overall survival (OS) in node-negative and node-positive breast cancer. Additional prognostic factors such as oncogenes, growth factors and secretory proteins have been investigated and appear to correlate with tumour behaviour with respect to differentiation, growth rate and metastatic pattern. However, there is still a need to identify more cellular and genetic parameters that will help define the complex biological profile of a breast tumour cell. More recently microarray analysis provides a tool for tumour subclassification (Perou *et al*, 2000) and neural networks combine the available information to provide cumulative and more informative predictions (De Laurentiis *et al*, 1999).

Among different biochemical markers that can be used for monitoring cancer, serine proteases attracted particular interest because of their role in degradation of the extracellular matrix (Tryggvason *et al*, 1987; Duffy, 1991) and stimulation of cell growth and angiogenesis. (Gottesman, 1990; Liotta *et al*, 1991). Accumulating data suggest that many members of the expanded human tissue kallikrein gene family are associated with malignancy (Diamandis *et al*, 2000a; Yousef and Diamandis, 2001). Prostate specific antigen (PSA; encoded by the KLK3 gene) is the best tumour marker for prostate cancer (Diamandis, 1998. Other members of the kallikrein family include human glandular kallikrein 2 (hK2), which is now an emerging tumour marker for prostate cancer (Kwiatkowski *et al*, 1998; Magklara *et al*, 1999). Among all other kallikreins, the following has been reported: prognostic value of KLK4, KLK7, KLK8 and KLK10 in ovarian cancer, diagnostic value of hK6 and hK10 in ovarian cancer and association of KLK10, KLK14 and KLK15 with testicular, breast and prostate cancer (Tanimoto *et al*, 1999; Underwood *et al*, 1999; Diamandis *et al*, 2000b; Yousef *et al*, 2000b, 2001a,b; Luo *et al*, 2001a,b; Magklara *et al*, 2001).

The human kallikrein gene 13 (KLK13), previously known as KLK-L4, is a newly identified member of the human kallikrein gene family that maps to chromosome 19q13. At the mRNA level, this gene is mainly expressed in testis, breast, prostate and salivary (Yousef *et al*, 2000a). The predicted protein structure has the conserved catalytic triad of serine protease, like the other members of this family. KLK13 was found to be down-regulated (at the mRNA level) in a preliminary set of 19 breast tumours. The objective of this study was to further investigate the relationship between KLK13 expression and other clinicopathological variables and DFS and OS using, univariate and multivariate analysis for a group of 173 breast cancer patients. We hypothesised KLK13 may be differentially expressed in breast cancer tissues and may have prognostic/predictive value.

## MATERIALS AND METHODS

### Study population

Included in this study were tumour specimens from 173 consecutive patients undergoing surgical treatment for primary breast carcinoma at the Department of Gynecological Oncology at the University of Turin, Turin, Italy. Diagnosis was confirmed by histopathology in all cases. Tumour tissues had been frozen in liquid nitrogen immediately after surgery. This study has been approved by the Institutional Review Board of the University of Turin. The patient ages ranged from 29 to 87 with a median of 58 years. Tumour sizes ranged from 0.1 to 15 cm with a median of 2.15 cm. Follow-up information (median follow-up period 80 months) was available for 163 patients, among whom 48 (29%) had relapsed and 42 (26%) died. The histological type and steroid hormone receptor status of each tumour as well as the number of positive axillary nodes were established at the time of surgery, as shown in Table 1

**Table 1:**
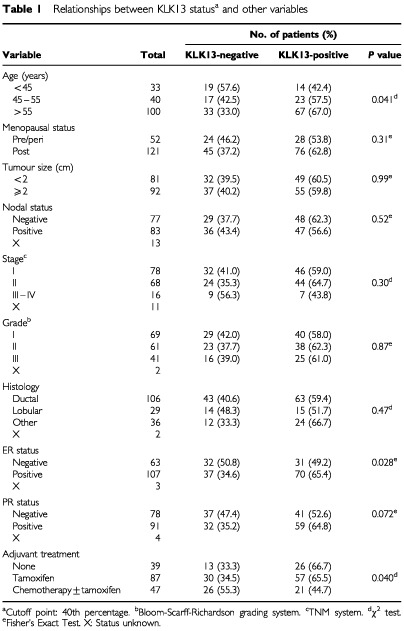
Relationships between KLK13 status^a^ and other variables

. Out of the 179 patients, 106 (61%) had ductal carcinoma, 29 (17%), lobular carcinoma and 36 (21%) had other histological types. Patients from all clinical stages (I – IV) were included in the study, with clinical staging determined according to the TNM classification system. Grading of tumours was done according to the Bloom-Scarff-Richardson grading system (Bloom and Richardson, 1957). Thirty-nine patients (24%) received no adjuvant treatment, 87 (50%) received tamoxifen, and 47 (27%) received chemotherapy with or without tamoxifen. Oestrogen receptor (ER) and progesterone receptor (PR) status was established as described by the European Organisation for Research and Treatment of Cancer (EORTC, 1980).

### Total RNA extraction and cDNA synthesis

Tumour tissues were minced with a scalpel, on dry ice, and transferred immediately to 2 ml polypropylene tubes. They were then homogenised and total RNA was extracted using Trizol^TM^ reagent (Gibco–BRL) following the manufacturer's instructions. The concentration and purity of RNA were determined spectrophotometrically. Two μg of total RNA were reverse-transcribed into first strand cDNA using the Superscript^TM^ preamplification system (Gibco–BRL). The final volume was 20 μl.

### Quantitative real-time PCR and continuous monitoring of PCR products

Based on the published genomic sequence of KLK13 (GenBank accession no. AF135024), two gene-specific primers were designed (L4-LF2: 5′-TGT ATG GCA TCG TCT CCT GG-3′ and L4-LR2: 5′-AGG TGG TGA TCT GGG CTC AT-3′). These primers spanned more than two exons to avoid contamination by genomic DNA.

Real-time monitoring of PCR reaction was done using the LightCycler^TM^ system (Roche Molecular Systems, Indianapolis, IN, USA) and the SYBR Green I dye, which binds preferentially to double stranded DNA. Fluorescence signals are proportional to the concentration of the product and are measured at the end of each cycle and immediately displayed on a computer screen, permitting real time monitoring of the PCR reaction (Wittwer *et al*, 1997). The reaction is characterised by the point during cycling when amplification of PCR products is first detected, rather than the amount of PCR product accumulated after a fixed number of cycles. The higher the starting quantity of the template, the earlier a significant increase in fluorescence is observed (Bieche *et al*, 1999). The threshold cycle is defined as the fractional cycle number at which fluorescence passes a fixed threshold above baseline (Bieche *et al*, 1998).

### Endogenous control

For each sample, the amount of the target and an endogenous control (β-actin, a housekeeping gene) were determined using a calibration curve (see below). The amount of the target molecule was then divided by the amount of the endogenous reference, to obtain a normalised target value.

### Standard curve construction

**Figure 1:**
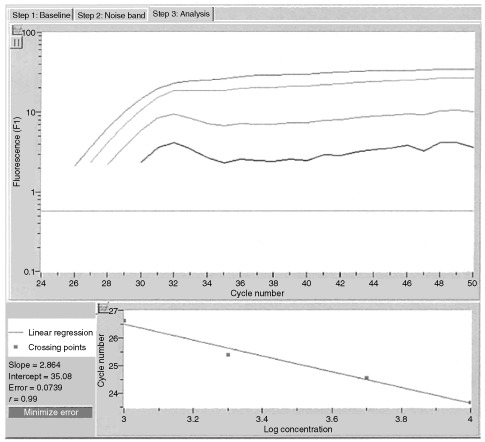
Quantification of KLK13 gene expression by real-time PCR. Top: A logarithmic plot of fluorescence signal above the noise level (horizontal line) *vs* cycle number, during amplification. Serial dilutions of a total RNA preparation from breast tissue were prepared and an arbitrary copy number was assigned to each sample according to the dilution factor. Bottom: The crossing points (cycle number) plotted against the log of copy number to obtain a standard curve. For details, see text.

Separate standard curves for actin and KLK13 were constructed using serial dilutions of total cDNA from healthy human breast tissue, purchased from Clontech, Palo Alto, CA, USA as described by Bieche *et al*, 1998, 1999. The standard curve samples were included in each run. The LightCycler^TM^ software automatically calculates the standard curve by plotting the starting dilution of each standard sample versus the threshold cycle, and the sample concentrations were then calculated accordingly (Figure 1

). Standards for both KLK13 and actin RNAs were defined to contain an arbitrary starting concentration, since no primary preparations exist. Hence, all calculated concentrations are relative to the concentration of the selected standard.

### PCR amplification

The PCR reaction was carried out on the LightCycler^TM^ system. For each run, a master mixture was prepared on ice, containing 1 μl of cDNA, 2 μl of LC DNA Master SYBR Green 1 mix, 50 ng of primers and 1.2 μl of 25 mM MgCl_2_. After the reaction mixture was loaded into the glass capillary tube, the cycling conditions were carried out as follows: initial denaturation at 95°C for 10 min, followed by 45 cycles of denaturation at 95°C for 0 s, annealing at 65°C for 5 s, and extension at 72°C for 25 s. The temperature transition rate was set at 20°C per second. Fluorescent product was measured by a single acquisition mode at 84°C after each cycle.

### Melting curve

For distinguishing specific from non-specific products and primer dimers, a melting curve was obtained after amplification by holding the temperature at 70°C for 30 s followed by a gradual increase of temperature to 98°C at a rate of 0.2°C/s, with the signal acquisition mode set at step, as described before (Woo *et al*, 1998). To verify the melting curve results, representative samples of the PCR products were run on 1.5% agarose gels, purified, and cloned into the pCR 2.1-TOPO vector (Invitrogen, Carlsbad, CA, USA) according to the manufacturer's instructions. The inserts were sequenced from both directions using vector-specific primers, with an automated DNA sequencer.

### Statistical analysis

Patients were subdivided into groups based on different clinical or pathologic parameters and statistical analyses were performed using SAS software (SAS Institute, Cary, NC, USA). A cutoff point equal to the detection limit (40th percentile) was used based on the ability of KLK13 to predict the DFS for the population studied. According to this cutoff, KLK13 expression was classified as positive or negative and associations between KLK13 status and other qualitative variables were analysed using the chi-squared (χ^2^) or Fisher's Exact Test, where appropriate. The analysis of differences in KLK13 values between groups of patients was performed with the nonparametric Mann–Whitney *U*-test or Kruskal-Wallis tests. In this analysis, KLK13 was used as a continuous variable. The cutoff value for tumour size was 2 cm. Lymph node status was either positive (any positive number of nodes) or negative. Age was categorised into three groups: less than 45 years, 45 to 55 years and greater than 55 years. Survival analyses were performed by constructing Kaplan-Meier DFS and OS curves (Kaplan and Meier, 1958) and differences between curves were evaluated by the log-rank test, as well as by estimating the relative risks for relapse and death using the Cox proportional hazards regression model (Cox, 1972). Cox analysis was conducted at both univariate and multivariate levels. Only patients for whom the status of all variables was known were included in the multivariate regression models, which incorporated KLK13 and all other variables for which the patients were characterised. The multivariate models were adjusted for KLK13 expression in tumours, patient age, nodal status, tumour size, grade, histological type and ER and PR status.

## RESULTS

### KLK13 expression in relation to other variables

The KLK13 arbitary mRNA levels range from 0 to 255 with a mean=14.1 s.e.=3.31 and median=0.22. An optimal cut-off point equal to the 40th percentile was defined with χ^2^ analysis based on the ability of KLK13 to predict the DFS for the population studied (data not shown). Table 1 depicts the distribution of KLK13 expression in relation to other prognostic factors such as menopausal status, tumour size, nodal status, tumour stage and grade, histological type, receptor status, and adjuvant therapy. The distribution of KLK13 values is right skew (data not shown). Sixty per cent of the samples had detectable expression of KLK13. KLK13 expression positivity was found more frequently in oestrogen receptor (ER) positive patients (*P*=0.028). KLK13 positivity was significantly higher in patients over the age of 55 years (*P*=0.041). Associations with menopausal status, tumour size, nodal status, histology and progesterone receptor (PR) status were not observed (*P*>0.05).

### Survival analysis

Out of the 173 patients included in this study, follow-up information was available for 163 patients, among whom 48 (29%) had relapsed and 42 (26%) died. Table 2a

**Table 2a:**
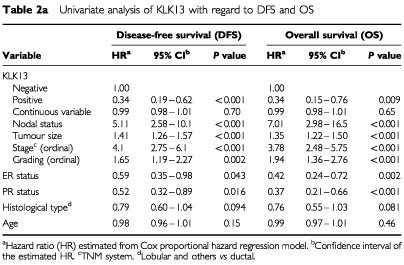
Univariate analysis of KLK13 with regard to DFS and OS

illustrates the strength between each clinicopathological variable and disease-free (DFS) and overall survival (OS). In univariate Cox regression analysis, positive KLK13 expression resulted in 66% increase in DFS and OS (*P*<0.01). As well, in multivariate Cox regression analysis, KLK13 expression was found to be a predictor of DFS and OS (with a hazard ratio (HR) of 0.41 and 0.46; *P*<0.001 and *P*=0.009, respectively). This regression model suggests there is approximately a 55–60% reduction in either the risk of relapse or death in patients with KLK13-positive tumours compared to those who are KLK13-negative. Kaplan-Meier survival curves (Figure 2

**Figure 2:**
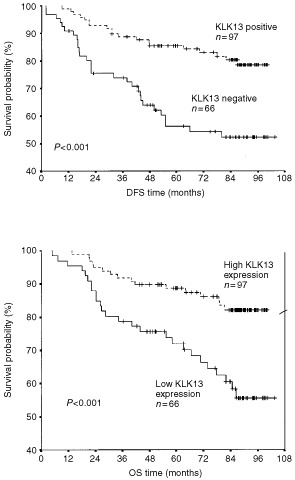
Kaplan-Meier analysis showing disease-free survival (DFS) and overall survival (OS) for patients with KLK13 positive and KLK13 negative tumours.

) also demonstrate that patients with KLK13 positive tumours have substantially higher DFS and OS (*P*<0.001) compared to those who are KLK13 negative.

In the multivariate analysis, Cox models were adjusted for nodal status, tumour grade, ER and PR status, histological type and age. In this analysis, KLK13 positivity, nodal status, and tumour size were found to be the strongest independent factors for DFS and OS (Table 2b

**Table 2b:**
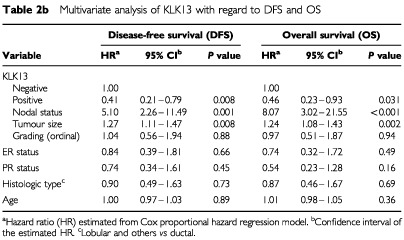
Multivariate analysis of KLK13 with regard to DFS and OS

). Tumour stage was not included in the multivariate models because it is a function of tumour size and nodal status. Table 3

**Table 3:**
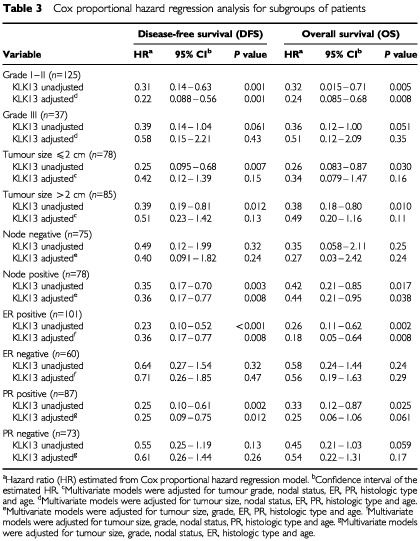
Cox proportional hazard regression analysis for subgroups of patients

illustrates Cox proportional hazard regression analysis for subgroups of patients stratified for nodal status, tumour grade and hormone receptor positivity. KLK13 was found to be a significant prognostic factor in the subgroup of patients who are node positive, oestrogen and progesterone receptor positive or those with grade I and II cancer. After adjusting for other known prognostic factors, KLK13 retained its independent prognostic value in all these subgroups of patients. The adjusted hazard ratios derived from this Cox regression analysis and related DFS and OS for these subgroups were 0.36 (*P*<0.001) and 0.18 (*P*=0.002), respectively, for the oestrogen receptor positive group; 0.36 (*P*=0.008) and 0.44 (*P*=0.038), respectively, for the node positive group; 0.22 (*P*=0.001) and 0.24 (*P*=0.008), respectively, for the Grade I–II group. The adjusted hazard ratio (HR) for PR positive patients and related DFS was 0.25 (*P*=0.012). For patients in the node positive and ER positive subgroup (*n*=51) the adjusted HR was 0.25 (*P*=0.006) and for the node positive and PR positive subgroup (*n*=46) the HR was 0.24 (*P*=0.008).

## DISCUSSION

Breast cancer therapy is based on the presence or absence of various clinical manifestations as well as a few biomarkers; therefore, identifying new prognostic and predictive markers will aid in optimal patient treatment. The classical prognostic markers for breast cancer, including lymph node status, tumour size and stage, have proven clinical value (Fitzgibbons *et al*, 2000). Many other potential prognostic markers have been identified, including steroid receptors, epidermal growth factor receptor (EGFR), p53, c-erbB2, Bcl-2, CEQ, CA15.3, CA27.29, cathepsin D and polyadeylate polymerase (ASCO, 1998, Fitzgibbons *et al*, 2000; Hamilton and Piccart, 2000; Nicholson *et al*, 1993, 1994; Norberg *et al*, 1996; Scorilas *et al*, 2000). However, only hormone receptor status is recommended for routine use by the American Society of Clinical Oncology and the College of American Pathologists Consensus Statement (Fitzgibbons *et al*, 2000). None of the remaining biomarkers have sufficient prognostic/predictive value by themselves. Some markers may have applications in particular cases, e.g. overexpression of c-erbB2 is considered to be an unfavourable prognostic indicator for both node-negative and node-positive patients (Scorilas *et al*, 1995, 1999b), and HER-2/neu is useful for patient selection for Herceptin therapy (Hamilton and Piccart, 2000). In this study, we demonstrate that KLK13 expression has an independent, favourable prognostic value in breast cancer.

Protease involvement in the development and progression of cancer has conventionally been considered to be unfavourable, since it may promote tumour invasion and metastasis. (Mignatti and Rifkin, 1993). Conversely, protease inhibitors are considered to be beneficial in inhibiting tumour progression (Kennedy, 1998). However, a new paradigm is emerging for several serine proteases in relation to prostate and testicular cancers. Human kallikrein 4 or ‘prostase’, was found to be expressed in the normal prostate but not in the prostate cancer cell lines DU-145 and PC-3 (Nelson *et al*, 1999). Testisin, a serine protease, was shown to be lost in testicular cancer through either loss of a gene (Hooper *et al*, 1999) or through promoter methylation (Boucaut *et al*, 2000). As well, transfection of human testicular cancer cells with a testisin cDNA reduced the tumour growth of xenografts of these cells in nude mice, suggesting a tumour suppressor function for testisin (Boucaut *et al*, 2000). Prostasin, another serine protease, has been implicated in normal prostate biology and is able to suppress prostate cancer invasion *in vitro* using DU-145 and PC-3 cell lines (Chen *et al*, 2001). Human kallikrein 10 (hK10) appears to inhibit tumour formation and the tumorigenic potential of breast cancer cell lines and is proposed to be a tumour suppressor (Goyal *et al*, 1998). In our studies of prognostic value of various kallikrein in cancer, we found down-regulation in breast cancer of KLK14 and in testicular cancer of KLK10. Furthermore, KLK8 and KLK9 expression are higher in ovarian cancer of better prognosis. Thus, recent literature suggests that serine proteases may be either favourable or unfavourable prognostic markers. When the substrates and physiological pathways of these proteases are delineated, a rational explanation of these findings may emerge.

Previously, KLK13 was found to be down-regulated in a subset of 19 breast tumours (Yousef *et al*, 2000a). KLK13 positivity is associated with a significantly large reduction in risk of relapse and death. However, the mechanism to explain the role of KLK13 in breast cancer aggressiveness is still unknown. KLK13 could mediate its role either by generating or activating breast cancer inhibitory factor(s) or by terminating the action of unfavourable factor(s). PSA has been well documented to be down-regulated in both prostate and breast cancer tissues (Yu *et al*, 1995, 1996, 1998), suggesting that it may, too, act as a favourable factor. Additional data suggest that PSA may be a tumour suppressor (Balbay *et al*, 1999), an inducer of apoptosis (Balbay *et al*, 1999), a negative regulator of cell growth (Lai *et al*, 1996), and an angiogenic inhibitor (Fortier *et al*, 1999). Human kallikrein 10, or the normal epithelial cell specific-1 (NES1), a serine protease, is down-regulated in breast and prostate cancer cell lines, and functions as a tumour suppressor (Goyal *et al*, 1998). Other proteases, such as Pepsinogen C and matrix metalloproteinase-9, have been found to be favourable indicators in breast cancer (Scorilas *et al*, 1999a, 2001).

An important factor predicting response to endocrine therapy is the presence of tumour cells with high ER and PR expression. Patients with ER-positive tumours have longer survival than patients with ER-negative tumours (2). Of patients whose tumours are positive for both ER and PR, 50 to 70% may benefit from endocrine therapy, while patients who are positive for ER only (Sedlacek and Horowitz, 1984), 40% will respond to endocrine therapy (Ravdin *et al*, 1992). Because endocrine therapy is generally associated with fewer side effects than chemotherapy, such as damage to the skeletal system (Bundred, 2001), increasing amount of research into new endocrine agents and drug development is rapidly growing (Buzdar, 2001). Since KLK13 is up-regulated by oestrogens and is a favourable prognostic marker in patients who are ER positive, we predict that KLK13 expression may have value for monitoring patients undergoing selective oestrogen receptor modulator (SERM) treatment. As patients with positive KLK13 expression have a 55-80% reduction in the risk of relapse or death, KLK13 may be used to monitor patients undergoing endocrine therapy for favourable outcome. In addition, we believe that patients who are ER positive but express low levels of KLK13 may not be responsive to hormonal therapy. Further experiments using different patient groups should be conducted to determine if KLK13 expression is associated with the response to endocrine therapy in breast cancer and to elucidate the possible clinical utility in KLK13 expression.

This study is the first to describe KLK13 as an independent favourable prognostic marker in breast cancer. Positive KLK13 expression is associated with a significantly larger increase in DFS and OS in both univariate and multivariate analyses and patients who are ER positive, node positive or have low grade tumours. KLK13 may potentially be a biomarker for identifying patients likely to benefit from hormonal treatment. Future studies should examine such a possible role of KLK13 in the management of patients with breast cancer.
